# Editorial: Unfolding the mechanism of hepatocyte injury of HBV precore and core promoter variants

**DOI:** 10.1097/HEP.0000000000000400

**Published:** 2023-04-11

**Authors:** Nikolaus Jilg, Thomas F. Baumert

**Affiliations:** 1 Massachusetts General Hospital, Boston, MA, USA; 2 Brigham and Women’s Hospital, Boston, MA, USA; 3 Harvard Medical School, Boston, MA, USA; 4 Université de Strasbourg, Inserm, Institut de Recherche sur les Maladies Virales et Hepatiques UMR_S1110, Strasbourg; 5 Service d’hépato-gastroentérologie, Hôpitaux Universitaires de Strasbourg, Strasbourg; 6 Institut hospitalo-universitaire (IHU), Université de Strasbourg, Strasbourg; 7 Institut Universitaire de France (IUF), Paris

Hepatitis B remains one of the major global causes for cirrhosis and hepatocellular carcinoma (HCC). Hence, even though effective vaccines and therapies for prevention and control of hepatitis B virus infection exist, a better understanding of the pathophysiology causing complications like acute liver failure, end-stage liver disease or HCC is a priority, particularly in view of those patients who currently do not benefit from available therapies, e.g., because they already had advanced disease with complications such as HCC at the time of diagnosis, or because they do not have access to adequate medical care. The risk for hepatitis B reactivation even after prolonged periods of inactive infection poses another problem, particularly for people with considerable immunosuppression^1^. Finally, while nucleos(t)ide analogs are standard of care for control of viral infection and prophylaxis of reactivation, they can only rarely induce cure.

There are several known risk factors for unfavorable outcomes of hepatitis B: these include perinatal transmission or transmission earlier in childhood which go along with a substantially increased risk of developing chronic hepatitis. High viral replication and highly elevated markers of liver inflammation as well as co-infection with hepatitis D virus lead to faster progression to cirrhosis. Hepatitis B surface antigen clearance on the other hand heralds a highly favorable prognosis and substantially reduces the risk for complications. Moreover, viral genotypes and defined viral mutations in the HBV core region were found to influence the course of the infection: the precore (PC) mutation G1896A gives rise to a stop codon thereby inhibiting production of HBV e-antigen (HBeAg) and has initially been found in patients with fulminant hepatitis.^2^ Furthermore, defined mutations in the basal core promoter (BCP), lead to decreased HBeAg levels, and result in increased viral replication in cell culture models of HBV infection.^3–5^ BCP mutant HBV was found to be associated with a higher cirrhosis risk.^6^ Moreover, PC and BCP mutations are significantly associated with hepatitis B reactivation as a complication of immunosuppression.^7^ Additionally, the mutations decrease the chance of response to therapy with peginterferon, and potentially to nucleos(t)ide analogs.^8,9^

The associations between PC and BCP mutations, worse clinical outcomes, and low or absent HBeAg led to hypotheses that HBeAg may induce at least partial immune tolerance towards HBV and thereby limit hepatic injury by the immune system. While these mutations have been associated with enhanced viral replication in cell culture models and patients, the genotype-phenotype relationship in vivo is not known. In particular, given their prevalence in different disease manifestations, it remains unclear whether these mutations result in liver damage in vivo.

To address these questions, Uchida and colleagues in Professor Kazuaki Chayama’s laboratory (Hiroshima University, Japan) in collaboration with Dr. T. Jake Liang (LDB, NIDDK, National Institutes of Health, USA) and others investigated in vivo infection of HBV PC and BCP variants in the uPA/SCID mouse model that lacks relevant parts of the immune system and is permissive for the transplantation of human hepatocytes.^10^ In the first series of experiments, they demonstrated that infection with a PC/BCP double mutant HBV (HBV-mt/mt) led to higher HBV DNA levels compared to a wildtype virus (HBV-wt). After about 5 weeks, the HBV-mt/mt infected animals showed rapid decline of HBV DNA in parallel with increase in human albumin reflecting loss of human hepatocytes suggesting a cytopathic effect on the engrafted human hepatocytes which was not evident in those mice infected with wildtype virus. Higher viral loads were also found in supernatants from cultures of human hepatocytes that were infected with HBV-mt/mt versus wildtype.

Next, the authors demonstrated that human liver chimeric mice infected with wildtype HBV and superinfected with HBV-mt/mt subsequently develop HBV DNA and human albumin decline as signs of cell death caused by the mutant virus. HBeAg levels were preserved at the time HBV DNA and albumin levels began to decrease demonstrating that hepatocellular injury was not prevented by the presence of HBeAg. Moreover, HBV-mt/mt infected mice showed considerably higher accumulation of HBV surface antigen (HBsAg) in hepatocytes, and the authors demonstrate colocalization of HBsAg with the ER in these mice. With this finding, Uchida and colleagues hypothesized that there may be a direct cytopathic effect linked to the endoplasmic reticulum (ER). This is supported by their transcriptomic experiments in that animals infected with the double mutant, but not wildtype HBV showed induction of genes that are part of the unfolded protein response (UPR). UPR is stress reaction of the ER following accumulation of unfolded or misfolded proteins that is characterized by countermeasures aimed at stopping production of misfolded proteins and restoration of normal translation, but eventually leads to apoptosis if these mechanisms are not successful. The investigators then used recombinant HBV constructs to study different combinations of the PC/BCP mutations compared to wildtype HBV (HBV-wt/wt, HBV-mt/mt, HBV-wt/mt, HBV-mt/wt) and demonstrate that in their mouse model, infection with the double-mutant and both single-mutant constructs led to higher viral loads followed by a decrease of hepatocyte viability compared to infection with wildtype.

What are the implications of the study for HBV pathogenesis and clinical outcomes? Mechanistically, liver injury and hepatocyte death in hepatitis B have been ascribed to the host’s immune response rather than to a direct cytopathic effect of the virus. Indeed, HBV is considered a “stealth virus”.^11^ Studies to further elucidate the underlying pathophysiology, however, have been hampered by the complex interplay of various viral and host factors present in patients. By using a model system that lacks the major components of adaptive immunity, Uchida et al. showed that in contrast to wild-type virus the variants result in hepatocyte injury in the absence of an adaptive immune response. These studies confirm and extend the phenotype of previous studies in primary human Tupaia hepatocytes having shown that the BCP variants induce apoptosis.^12^ Uchida and colleagues elucidated the mechanism of this finding by showing a direct association between accumulation of HBsAg in the ER and an increase of the unfolded protein response and markers of cell death. Since overexpression of HBsAg has been shown to be associated with increased markers of the UPR and apoptosis in cell-based models as well as in tissues of HBV-infected patient livers^13^, it is likely that this mechanism occurs by infection of the variants in vivo. Nevertheless, it cannot be excluded that additional mechanisms mediating cell death may also be at play.

Clinically, it remains intriguing that the PC and BCM variants have been associated with various disease manifestations ranging from asymptomatic carriers to fulminant hepatitis. The association of PC and BCM variants with more aggressive disease in patients, suggest that variant-induced cell death may contribute to the clinical outcome of patients infected with the variants. However, given the range of disease manifestations, it is likely that host-specific factors additionally play a role in disease biology. Experiments with hepatocytes from different donors with different genetic backgrounds could address this question in a next step.

Hepatocyte injury seen in the immunocompromised mouse model may also mimic features of HBV reactivation seen in patients who are immunosuppressed either due to relevant comorbidity (e.g., hematologic malignancy, status post transplantation, advanced HIV infection), or immunosuppressive medication, the latter becoming more prevalent in view of expanding indications for chemotherapy, organ and stem cell transplantations, and immunosuppressive therapy of malignancies and autoimmune diseases (e.g., anti-CD-20 antibody, and many others) as well as longer survival of these patients. Absent control of viral infection by adaptive and innate immune responses in these patients may result in a similar phenotype as observed by Uchida et al. Chen et al., for instance, found deterioration of liver function in most participants in a cohort of HBV carriers after hematopoietic stem cell transplantation, and a significantly increased risk for hepatitis if HBV with PC or BCP mutations was present in those who were transplanted.^14^ The present study suggests that a status of high viral replication, e.g., during hepatitis B reactivation in the setting of immunosuppression, may carry an increased risk for hepatocyte injury from direct cytopathogenicity of HBV. Prophylaxis against or early treatment during reactivation to efficiently and rapidly suppress viral replication in patients with HBV reactivation may therefore be important to protect against liver damage.

There are also limitations as the authors raise in the discussion. The model and the current results are based on a limited number of experiments and replicates which is related to the challenge to create the human liver chimeric mice. Only one HBV genotype was used. Furthermore, since the strains were from two different patients, other mutations beyond the PC and BCM mutations may play a functional role in the observed phenotypes. Therefore, the investigation of genotype-specific or viral co-factors would be another field of study.

In summary, Uchida et al. made an elegant contribution to the field by answering the question on how the PC and BCP variants can result in liver injury and more aggressive disease in patients. By providing novel mechanistic insight for clinical translation, the findings of Uchida et al. may also be useful in the quest to eliminate these variants by next generation strategies for HBV cure.

## Figures and Tables

**Figure 1:**
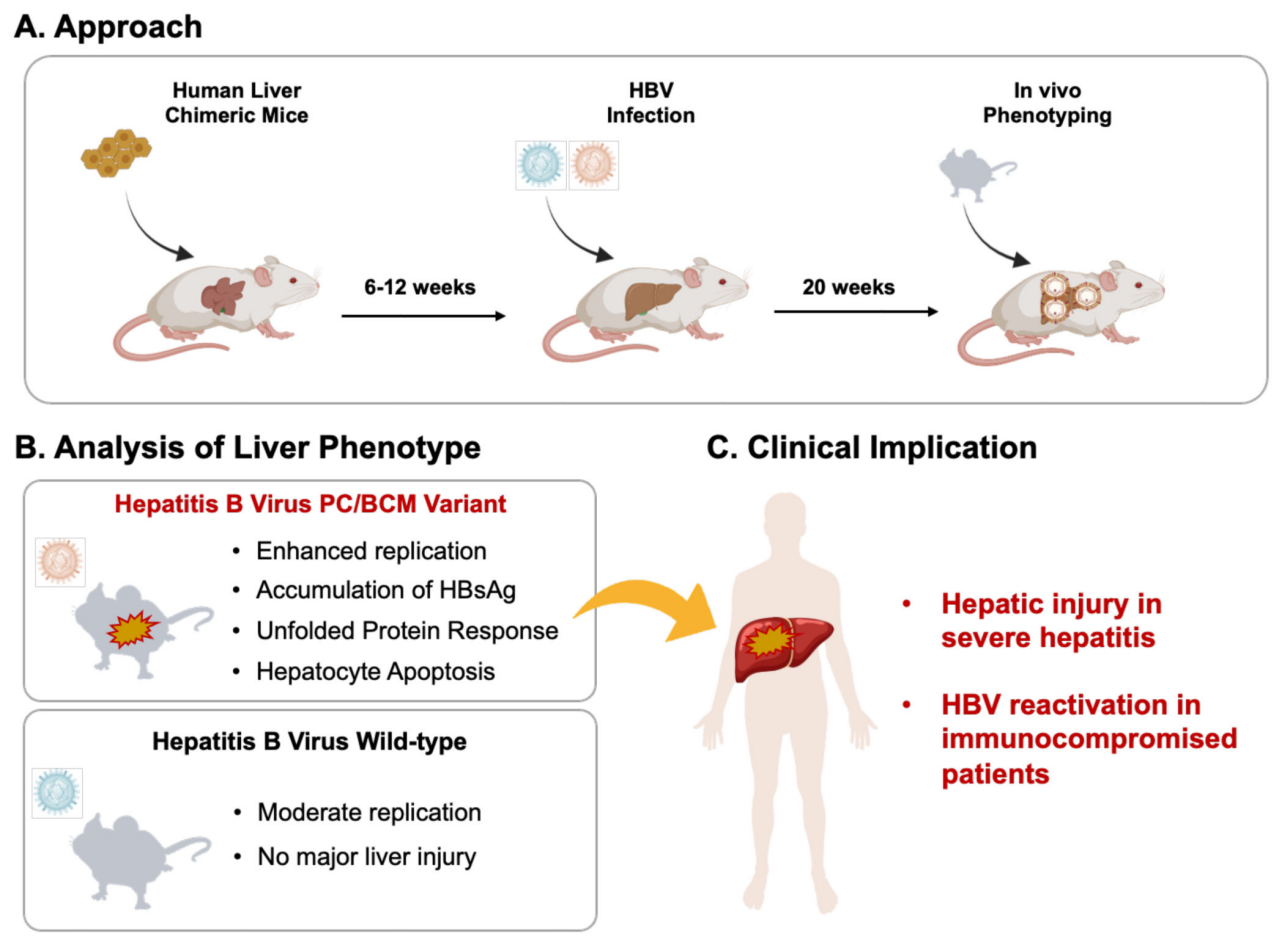
Model of in vivo phenotype of PC and BCP variants accoding to findings by Uchida et al. Human liver chimeric mice were infected with PC/BCP variant or wild-type HBV and the liver phenotype analysed by different approaches (A). Compared to wild-type virus, the variants resulted in enhanced replication, HBs accumulation, activation of the unfolded protein response and cell death (B). These findings may explain the clinical phenotype associated with the variants in patients (C). Abbreviations: HBV – hepatitis B virus; PC – precore mutation; BCP – basal core promoter mutations.
